# Engaging the Community: CASP Special Interest Groups

**DOI:** 10.1002/prot.26833

**Published:** 2025-04-30

**Authors:** Arne Elofsson, Rachael C. Kretsch, Marcin Magnus, Gaetano T. Montelione

**Affiliations:** ^1^ Department of Biochemistry and Biophysics and Science for Life Laboratory Stockholm University Solna Sweden; ^2^ Biophysics Program Stanford University Stanford California USA; ^3^ Department of Molecular and Cellular Biology Harvard University Cambridge Massachusetts USA; ^4^ Department of Chemistry and Chemical Biology Center for Biotechnology and Interdisciplinary Studies Rensselaer New York USA

**Keywords:** blind challenges, collaboration, community engagement, online community, open science, scientific exchange, structure prediction, webinar series

## Abstract

The Critical Assessment of Structure Prediction (CASP) brings together a diverse group of scientists, from deep learning experts to NMR specialists, all aimed at developing accurate prediction algorithms that can effectively characterize the structural aspects of biomolecules relevant to their functions. Engagement within the CASP community has traditionally been limited to the prediction season and the conference, with limited discourse in the 1.5 years between CASP seasons. CASP special interest groups (SIGs) were established in 2023 to encourage continuous dialogue within the community. The online seminar series has drawn global participation from across disciplines and career stages. This has facilitated cross‐disciplinary discussions fostering collaborations. The archives of these seminars have become a vital learning tool for newcomers to the field, lowering the barrier to entry.

The Critical Assessment of Structure Prediction (CASP) is a community‐wide experiment to assess the state‐of‐the‐art in structure prediction. The community encompasses diverse scientists who develop new structure prediction algorithms, conduct experiments to solve new structures, or assess, compare, and contrast prediction algorithms. CASP operates on a 2‐year cycle, characterized by intense discussions and engagement leading up to each biennial conference and significantly fewer activities in between. Although this structure has proven effective, as demonstrated by CASP's ongoing presence in the field and the community's enthusiastic participation, it presents particular challenges, particularly for newcomers who may have limited resources and face difficulties navigating the long intervals between meetings.

Recognizing these challenges, a proposal emerged at CASP15 to enhance community engagement and foster continuous dialogue. This led to the formation of special interest groups (SIGs) to encourage in‐depth discussions among members within the broader CASP community. Three SIGs were established: CASP‐AI, CASP‐RNA, and CASP‐Ensemble. Each group was designed to cater to specific areas of interest, yet they shared a common structure that facilitated regular interactions. They held monthly or biweekly online meetings to enable ongoing collaboration and knowledge sharing, which are more frequent, accessible, inclusive, and environmentally friendly than in‐person alternatives [1, 2, 3].

The establishment of these SIGs has transformed participants' experiences. Active discussions within each group have provided a platform for sharing insights and advancements in their respective fields and have helped bridge gaps for newer members and between disciplines, offering a supportive environment to learn, connect with seasoned professionals, and form collaborations toward progressing and expanding the goals of CASP.

This overview delves deeper into our experiences with each SIG, highlighting key discussions, innovative projects, and their overall impact on community engagement. This ongoing dialogue has enriched our collective understanding and paved the way for a more inclusive and dynamic CASP community that thrives on collaboration and shared knowledge.

## 1 CASP AI SIG

The inaugural *CASP Artificial Intelligence Special Interest Group* (*CASP‐AI SIG*) meeting occurred on January 11, 2023. This initial gathering sparked a series of monthly meetings that continued through the CASP16 season. Generally, the structure of these meetings featured informative presentations followed by engaging discussions. On several occasions, the focus shifted toward thematic talks, allowing a deeper exploration of specific topics within the CASP‐AI community. Most presentations were recorded and made publicly available, providing valuable resources for participants and interested individuals. To access these recordings, please refer to the homepage https://casp.bioinfo.se/ where links to all recorded seminars are provided.

Following CASP15, interest in the meetings surged, attracting approximately 100 participants per session and resulting in hundreds of views on YouTube. Among the various seminars, one of the standout presentations was delivered by Nazim Bouatta, who discussed the innovative process of retraining OpenFold, which resonated strongly within the community and garnered significant attention.

However, as we transitioned into 2024, a noticeable participation decline was observed. Attendance at most seminars dropped to around 50 participants, prompting concerns about the group's sustainability and engagement. Additionally, a few planned workshops were regrettably canceled at the last minute because of unforeseen circumstances, such as illness.

There are ambitious plans to revitalize the CASP‐AI SIG in 2025. One important consideration is the potential for a renewed focus on issues directly related to the CASP initiative, given numerous other activities with a similar profile within the community. Emphasizing CASP‐related topics may foster greater engagement, reignite interest among participants, and strengthen the SIG's collaborative spirit that characterized the early days. With a comprehensive strategy for the upcoming year, we hope to recapture the enthusiasm and participation levels experienced during our initial meetings, ensuring the CASP‐AI SIG remains a vibrant and valuable forum for discussion and innovation.

## 2 CASP NA SIG

The *Nucleic Acid Special Interest Group* (*CASP NA SIG*) is a group of nucleic acid enthusiasts united by the common goal of producing predictive algorithms for nucleic acid structure prediction. We aim to meet virtually regularly to discuss the state of new developments in our community, fostering an active discussion that brings together the diverse expertise necessary to tackle this problem.

Community members include those conducting research towards this goal, including but not limited to obtaining experimental data, building training and testing sets, obtaining RNA sequence alignments, predicting RNA or DNA secondary structure, predicting RNA 3D structure (contact prediction, fragment assembly, molecular dynamics (MD), structure scoring, deep learning), predicting ligand interactions, predicting protein interactions, predicting RNA–RNA interactions, and predicting ensembles of nucleic acid structures.

Between the CASP15 and CASP16 prediction seasons, we addressed a wide range of topics, inviting speakers from a diverse range of disciplines. To disseminate easy‐to‐use prediction algorithms, a key goal of the community was to close the accuracy gap between humans and automated approaches. Towards this goal, we discussed new innovative deep learning and other automated approaches in the tertiary and secondary structure spaces, databases for structural information, experimental errors to watch out for within our structural databases, and how high‐throughput biochemical data could be utilized in prediction. CASP NA SIG is also a platform to draw attention to new prediction challenges. Experimental structural biologists provided insights about the critical structural features of nucleic acid– protein complexes, and we explored how computational structural biologists aim to predict these structures. Finally, we discussed the functional relevance of RNA dynamics and how simulation methods may tackle predicting these energy landscapes; an area we are excited to see CASP explore more in the CASP Ensemble SIG.

The CASP NA SIG is a venue to learn about and discuss the latest research and brainstorm future directions for the nucleic acid structure prediction field. Archives of seminar recordings, https://www.youtube.com/@CASPRNASIG, are a resource that complements other online educational resources [4, 5]. Moving forward, we aim to enhance the vibrancy of our community through initiatives such as announcements in our biweekly emails and facilitated discussions aimed at fostering collaboration and reframing our goal‐posts for structure prediction accuracy.

## 3 CASP Ensemble SIG

Recognizing that biomolecules do not have static structures, but rather adopt a distribution of conformational states, the *CASP SIG on Ensembles of Conformations* aims to evaluate whether a critical assessment of predictions of conformational ensembles or multiple conformational states of biomolecules is feasible. The SIG considers developments in structural and computational biology that are anticipated in the next 2–5 years, and aims to define protocols for assessing the accuracy of multiple or ensemble model predictions.

Conformational ensembles range from simple two‐state systems, such as sidechain flips between rotamer conformations and enzyme lids in open vs. closed states, to systems adopting wide ranges of conformational states, as observed for intrinsically disordered proteins (IDPs) or intrinsically disordered regions of proteins (IDRs). Speakers in 2023–2024 have covered a range of topics relevant to methods for modeling conformational ensembles including shallow multiple‐sequence alignment (MSA) sampling (M. Feig, J. Meiler, S. Wodak, L. Porter, H. Waynent‐Steele), AlphaFold‐sample using multiple network parameters and dropouts (B. Wallner), AlphaFlow (B. Jing), and MD simulations (G. Palermo) with applications to various systems including IDPs (M. Blackledge, W. Vranken), fold‐flipping proteins (J. Orban, L. Porter, H. Wayment‐Steele), domain‐linker‐domain systems (B. Donald, T. Oas), and RNAs (R. Das, R. Kretsch). We have discussed strengths, weaknesses, and complementarity of various experimental methods for characterizing conformational dynamics and multiple conformational states, including X‐ray crystallography (J. Fraser, S. Wankowicz), NMR (M. Blackledge, W. Vranken), SAXS (S. Tsutakawa), and cryo‐electron microscopy (W. Chiu). We have also discussed formats and ontologies for representing multiple conformational states of proteins in Protein Data Bank coordinate files (G. Montelione, S. Wankowicz). Video recordings of these presentations are available on the *CASP SIG on Conformational Ensembles* YouTube channel, https://www.youtube.com/@CASPSIGConformationalEnsembles/.

The *CASP SIG on Conformational Ensembles* guided the design of the corresponding Ensembles experiment in CASP16 and spawned important questions that continue to be debated in the evolving field. Some of these include:

- How do we define a conformational state?
- How should we represent multi‐state conformational models in the PDB?
- Can we assess predicted multi‐state conformational models against experimental data? or against statistical models derived from experimental data?
- What is the impact of training memorization in AI‐based modeling?
- How can we combine/compare AI models of ensembles with MD methods?
- How can we combine experimental data and predicted models?

Although the initial online SIG Zoom meetings included more than 100 participants, the typical attendance was ~50 people over the months. In 2025, we plan to revisit some of the same topics covered in 2024, as the field has moved rapidly and several structural biology and computer science research groups have made significant advances.

## Author Contributions

**Arne Elofsson:** conceptualization, writing – original draft, writing – review and editing. **Rachael C. Kretsch:** conceptualization, writing – original draft, writing – review and editing. **Marcin Magnus:** writing – review and editing, writing – original draft, conceptualization. **Gaetano T. Montelione:** conceptualization, writing – original draft, writing – review and editing.
